# Tailoring the Extent of Lymphadenectomy for Esophageal Squamous Cell Carcinoma: Insights From a Comparative Study of Neoadjuvant Chemo‐Immunotherapy and Surgery Cohort

**DOI:** 10.1111/1759-7714.70297

**Published:** 2026-05-07

**Authors:** Kunheng Du, Hongwei Lin, Chenyi Xie, Ziyu Ning, Jiahui Chen, Changhong Liang, Haiyu Zhou, Zaiyi Liu, Yihuai Hu

**Affiliations:** ^1^ Department of Radiology, Guangdong Provincial People's Hospital (Guangdong Academy of Medical Sciences) Southern Medical University Guangzhou China; ^2^ Guangdong Provincial Key Laboratory of Artificial Intelligence in Medical Image Analysis and Application Guangzhou China; ^3^ Department of Cardiothoracic Surgery The First Affiliated Hospital of Guangzhou University of Chinese Medicine Guangzhou China; ^4^ Department of Thoracic Surgery, Guangdong Provincial People's Hospital (Guangdong Academy of Medical Sciences) Southern Medical University Guangzhou China; ^5^ Department of Pathology, Guangdong Provincial People's Hospital (Guangdong Academy of Medical Sciences) Southern Medical University Guangzhou Guangdong China

**Keywords:** esophageal squamous cell carcinoma, lymph node dissection, neoadjuvant therapy, prognosis

## Abstract

**Background:**

Lymph node dissection is crucial for accurate staging and prognosis assessment in esophageal squamous cell carcinoma (ESCC). While sufficient examined lymph nodes (ELNs) are generally linked to better outcomes, how ELN count affects prognosis under immunotherapy remains unclear.

**Methods:**

This study analyzed 621 ESCC patients who underwent R0 resection with or without neoadjuvant chemo‐immunotherapy (NACI). Patients were stratified into surgery alone (SA) (*n* = 451) and NACI (*n* = 170) groups. Propensity score matching balanced baseline characteristics. The relationship between ELN count and overall survival was analyzed using Cox regression models. Single‐cell RNA and T‐cell receptor sequencing were performed on paired tumor and lymph node samples to elucidate underlying immune mechanisms.

**Results:**

In the NACI cohort, both insufficient (ELN ≤ 23) and excessive (ELN > 31) lymph node resection were independent risk factors for worse overall survival (ELN ≤ 23: HR = 2.29, 95% CI 1.11–4.71, *p* = 0.024; ELN > 31: HR = 2.71, 95% CI 1.17–6.26, *p* = 0.020). However, the SA cohort derived continuous survival benefit from higher ELN counts. Single‐cell sequencing revealed that NACI enriched a population of activated, tumor‐reactive cytotoxic T cells within metastasis‐negative lymph nodes.

**Conclusions:**

The optimal ELN count is contingent on treatment strategy. For SA, a more extensive lymphadenectomy improves survival. However, for NACI, a “Goldilocks” principle applies—an ELN count between 24 and 31 balances accurate staging with the preservation of antitumor immunity, advocating for function‐preserving, personalized surgery in the immunotherapy era.

## Graphical abstract

In ESCC, the prognostic impact of examined lymph node (ELN) count was treatment‐dependent. Surgery alone showed continuous benefit with increasing ELNs, whereas after neoadjuvant chemoimmunotherapy, both insufficient and excessive ELN counts predicted worse survival. Excessive dissection may remove metastasis‐negative lymph nodes enriched with activated tumor‐specific cytotoxic T cells.

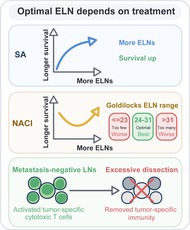

## 1 Introduction

Esophageal squamous cell carcinoma (ESCC) remains a major global health burden, particularly in Eastern Asia. Despite advances in multimodal treatment, prognosis remains poor, largely because many patients present with locoregionally advanced disease [1, 2]. Lymph node involvement is common and remains a major determinant of TNM staging, treatment stratification, and long‐term survival [3].

Lymph node sampling or dissection plays a crucial role in accurate lymph node staging and is also therapeutically significant for eliminating potential metastatic foci [3]. The number of examined lymph nodes (ELNs) is a core parameter for assessing the thoroughness of lymph node dissection and the accuracy of pathological staging, and a higher ELN count is significantly associated with improved patient survival, potentially attributable to more thorough tumor debulking and more precise staging‐guided treatment [4, 5].

With the widespread application of immune checkpoint inhibitors (ICIs) in the treatment of ESCC, the clinical value of excessive lymph node dissection is being questioned [6]. A significant proportion of patients achieve “pathological downstaging” after neoadjuvant immunotherapy, among whom a considerable proportion are staged N0 (no lymph node metastasis) [7, 8, 9]. In this context, the necessity of systematic lymph node dissection is debatable. Multiple studies have shown a nonlinear relationship between ELN and survival rate in ESCC patients receiving neoadjuvant therapy [6, 10].

However, the “optimal threshold” for the number of ELNs in ESCC remains controversial, with significant variations in the recommended ELN range across different guidelines and studies. Because nodal staging in the 8th AJCC TNM system is based on the number of metastatic lymph nodes, an adequate lymph node harvest is essential for accurate pathologic staging. NCCN Clinical Practice Guidelines have suggested that examination of more than 15 nodes improves nodal classification accuracy, and recent studies support a target of at least 14 nodes [4, 11, 12, 13, 14]. However, the optimal ELN threshold remains to be explored in the context of neoadjuvant immunotherapy.

This study aims to provide new evidence for optimizing postoperative staging and guiding adjuvant therapy in ESCC by defining the appropriate extent of lymph node dissection with or without ICI treatment. We analyzed a defined patient series to investigate this question and ultimately improve patient outcomes.

## 2 Methods

### 2.1 Study Population and Study Design

**FIGURE 1:**
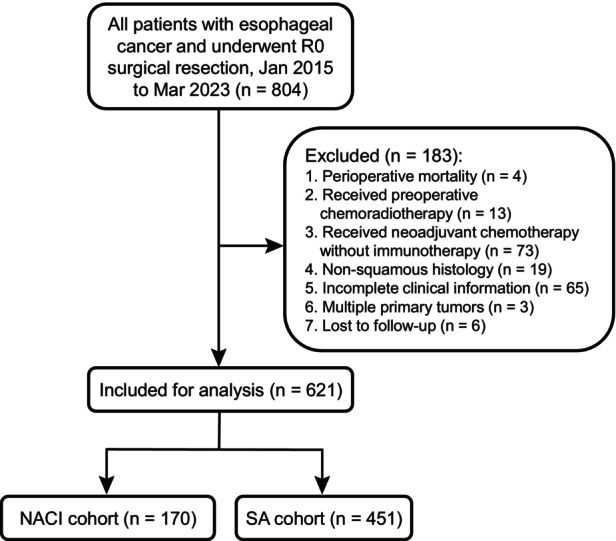
The flowchart of patient enrollment.

We retrospectively included patients with esophageal carcinoma who underwent R0 resection at Guangdong Provincial People's Hospital between January 2015 and March 2023, with or without neoadjuvant chemo‐immunotherapy (NACI). Exclusion criteria were perioperative mortality, preoperative chemoradiotherapy, neoadjuvant chemotherapy without immunotherapy, nonsquamous histology, incomplete clinical data, multiple primary tumors, and loss to follow‐up. Of 804 screened patients, 621 were eligible (Figure 1).

### 2.2 Treatment and Follow‐Up

Patients in the NACI group received 2–4 cycles of a PD‐1 inhibitor plus platinum‐based chemotherapy combined with docetaxel or another taxane every 3 weeks. Dose modifications were made according to general condition and liver and renal function. PD‐1 inhibitors included camrelizumab, nivolumab, tislelizumab, toripalimab, sintilimab, and pembrolizumab, each given intravenously at 200 mg every 3 weeks. Response was usually assessed after two cycles, and surgery was performed 4–6 weeks after the last cycle.

All patients underwent PET‐CT for preoperative staging, and clinical TNM stage was defined according to imaging findings. Patients who underwent surgery alone (SA) were mainly those with early‐stage disease (cT1–2/N0–1/M0), those who declined neoadjuvant therapy, or those with relative contraindications to systemic treatment. Esophagectomy was performed by minimally invasive or open approaches using the Ivor Lewis or McKeown procedure, with two‐field or three‐field lymphadenectomy.

The final follow‐up date was December 2024. Patients were followed up in the outpatient clinic every 3 months during the first 2 postoperative years and every 6 months thereafter. Long‐term survival data were obtained through telephone interviews, outpatient visits, and death registration records. Median follow‐up was 38.63 months (95% CI, 34.87–42.07).

### 2.3 Data Analysis

Patients were classified into the NACI and SA groups. To reduce confounding, propensity score matching (PSM) was performed using age, sex, tumor location, tumor differentiation, p/ypT stage and p/ypN stage, with 1:2 nearest‐neighbor matching and a caliper of 0.2 [15]. Unmatched patients were excluded. Overall survival (OS) was analyzed using the Kaplan–Meier method with the log‐rank test. Associations between clinicopathological variables and OS were evaluated using univariate and multivariable Cox proportional hazards models.

Analyses were performed in *R* version 4.3.2. Categorical variables were compared using the *χ* ^2^ test or Fisher's exact test, and continuous variables using the Student's *t*‐test or Mann–Whitney *U* test, as appropriate. Hazard ratios (HRs) with 95% confidence intervals were presented in forest plots. All tests were two‐sided, and *p* < 0.05 was considered statistically significant.

### 2.4 Tissue Acquisition and Single‐Cell RNA and V(D)J Sequencing

Three patients were included from each of the SA and NACI groups, and for each patient, one metastasis‐negative lymph node and one matched tumor tissue sample were collected. The majority of each tumor tissue and one half of each lymph node were subjected to histopathological evaluation by experienced pathologists, which confirmed the tumor tissues as ESCC and the lymph nodes as metastasis‐free. The remaining tissues were used for single‐cell RNA sequencing (scRNA‐seq) and single‐cell V(D)J sequencing. Details are provided in the Supporting Information.

## 3 Results

### 3.1 Baseline Characteristics of Patients

**FIGURE 2:**
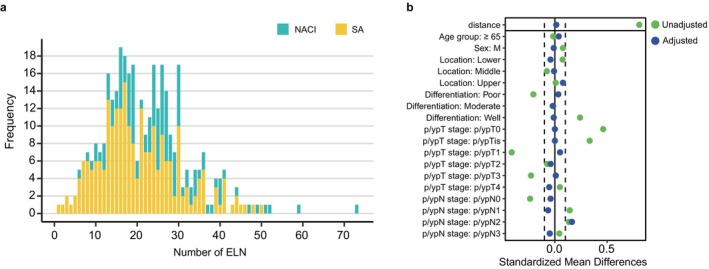
The distribution of ELN and comparison of variable balance before and after matching. (a) Bar graph showing the distribution of ELN in ESCC patients; (b) Love plot comparing the balance of variables before and after adjustment by PSM, with vertical dashed lines representing the threshold of ±0.1.

A total of 621 patients with ESCC were included, with a median OS of 55.6 months (95% CI, 47.2–74.2). Among them, 170 (27.4%) received NACI and 451 (72.6%) underwent SA. As shown in Table 1, the two groups did not differ significantly in age, sex, tumor location, surgical approach, or number of positive lymph nodes (PLNs) (*p* > 0.05). Patients in the NACI group had more advanced preoperative clinical stages, a higher proportion of well‐differentiated tumors, earlier pathological stages postoperatively, and a significantly higher number of lymph nodes dissected (Figure 2a, Table 1).

**TABLE 1:** Baseline characteristics of patients with esophageal squamous cell carcinoma before and after PSM.

Variable	Unmatched			Matched		
	NACI (*N* = 170)	SA (*N* = 451)	*p*	NACI (*N* = 121)	SA (*N* = 235)	*p*
Age (median [IQR])	60 [55–66]	61 [55–67]	0.489	61 [55–68]	61 [55–67]	0.504
Age group (%)			0.802			0.609
< 65	114 (67.06)	309 (68.51)		81 (66.94)	165 (70.21)	
≥ 65	56 (32.94)	142 (31.49)		40 (33.06)	70 (29.79)	
Sex (%)			0.482			1
Male	141 (82.94)	361 (80.04)		103 (85.12)	201 (85.53)	
Female	29 (17.06)	90 (19.96)		18 (14.88)	34 (14.47)	
ELN (median [IQR])	24 [18–30]	18 [13–26]	**< 0.001**	25 [18–30]	19 [14–26]	**< 0.001**
PLN (median [IQR])	0 [0–1]	0 [0–1]	0.365	1 [0–2]	1 [0–2]	0.823
Differentiation (%)			**< 0.001**			0.836
Poor	30 (17.65)	115 (25.50)		18 (14.88)	33 (14.04)	
Moderate	112 (65.88)	302 (66.96)		83 (68.60)	168 (71.49)	
Well	28 (16.47)	34 (7.54)		20 (16.53)	34 (14.47)	
Location (%)			0.672			0.661
Lower	71 (41.76)	172 (38.14)		50 (41.32)	103 (43.83)	
Middle	79 (46.47)	227 (50.33)		56 (46.28)	110 (46.81)	
Upper	20 (11.76)	52 (11.53)		15 (12.40)	22 (9.36)	
cT stage (%)			**< 0.001**			**0.001**
cT1	9 (5.29)	83 (18.40)		3 (2.48)	35 (14.89)	
cT2	86 (50.59)	144 (31.93)		57 (47.11)	80 (34.04)	
cT3	70 (41.18)	219 (48.56)		58 (47.93)	118 (50.21)	
cT4	5 (2.94)	5 (1.11)		3 (2.48)	2 (0.85)	
cN stage (%)			**< 0.001**			**< 0.001**
cN0	24 (14.12)	294 (65.19)		17 (14.05)	111 (47.23)	
cN1	83 (48.82)	90 (19.96)		57 (47.11)	74 (31.49)	
cN2	60 (35.29)	63 (13.97)		46 (38.02)	46 (19.57)	
cN3	3 (1.76)	4 (0.89)		1 (0.83)	4 (1.70)	
cTNM stage (%)			0.608			0.521
I	2 (1.18)	7 (1.55)		1 (0.83)	7 (2.98)	
II	61 (35.88)	181 (40.13)		44 (36.36)	79 (33.62)	
III	104 (61.18)	259 (57.43)		75 (61.98)	145 (61.70)	
IV	3 (1.76)	4 (0.89)		1 (0.83)	4 (1.70)	
p/ypT stage (%)			**< 0.001**			0.933
p/ypT0	30 (17.65)	0 (0.00)		0 (0.00)	0 (0.00)	
p/ypTis	17 (10.00)	0 (0.00)		0 (0.00)	0 (0.00)	
p/ypT1	22 (12.94)	121 (26.83)		21 (17.36)	38 (16.17)	
p/ypT2	37 (21.76)	112 (24.83)		37 (30.58)	77 (32.77)	
p/ypT3	63 (37.06)	217 (48.12)		63 (52.07)	119 (50.64)	
p/ypT4	1 (0.59)	1 (0.22)		0 (0.00)	1 (0.43)	
p/ypN stage (%)			0.055			0.510
p/ypN0	93 (54.71)	300 (66.52)		54 (44.63)	113 (48.09)	
p/ypN1	47 (27.65)	96 (21.29)		38 (31.40)	78 (33.19)	
p/ypN2	25 (14.71)	45 (9.98)		25 (20.66)	34 (14.47)	
p/ypN3	5 (2.94)	10 (2.22)		4 (3.31)	10 (4.26)	
p/ypTNM stage (%)			**< 0.001**			0.957
0	13 (7.65)	0 (0.00)				
I	2 (1.18)	4 (0.89)		2 (1.65)	4 (1.70)	
II	48 (28.24)	177 (39.25)		47 (38.84)	86 (36.60)	
III	102 (60.00)	260 (57.65)		68 (56.20)	135 (57.45)	
IV	5 (2.94)	10 (2.22)		4 (3.31)	10 (4.26)	
Surgical approach (%)			0.308			0.282
Minimally invasive	139 (81.76)	350 (77.61)		98 (80.99)	177 (75.32)	
Open	31 (18.24)	101 (22.39)		23 (19.01)	58 (24.68)	

*Note:* Bold values indicate statistical significance (*p* < 0.05).

To balance the disease burden and other clinical characteristics between the two groups of patients at the time of surgery, we included variables such as p/ypT stage, p/ypN stage, differentiation, tumor location, sex, and age for PSM. A total of 356 patients were matched. After matching, the two groups were better balanced with respect to these variables (Figure 2b; Table 1).

### 3.2 Relationship Between ELN Count and Prognosis

To assess the association between ELN count and prognosis, we performed an exploratory threshold‐scanning analysis in the matched NACI and SA cohorts. In each cohort, patients were repeatedly dichotomized at different ELN values, and the HR for patients above each threshold was calculated relative to those at or below it. Candidate thresholds were defined according to the ELN distribution and the requirement for adequate sample size in both subgroups. The feasible threshold ranges were 13–39 in the NACI cohort and 6–40 in the SA cohort.

**FIGURE 3:**
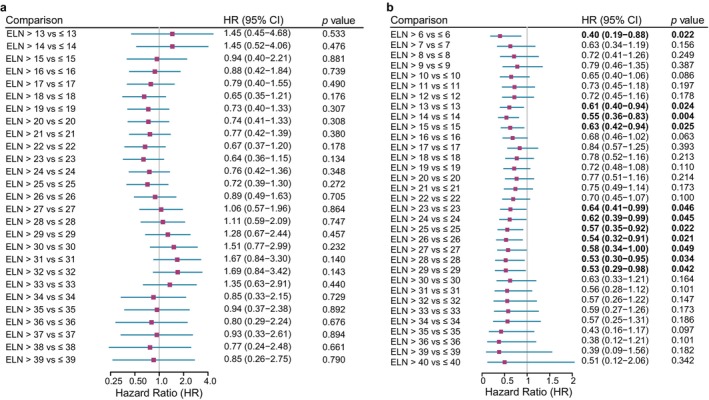
Forest plot of HR from Cox regression analysis stratified by ELN count. (a) NACI group; (b) SA group. HR (95% CI) and *p* value indicate the relative hazard of death for each category compared with the reference category. An HR greater than 1 indicates a higher risk.

In the NACI cohort, the group above the threshold gradually shifted toward a protective effect as the ELN cutoff increased. The lowest HR and smallest *p* value were observed at ELN = 23. With further increases in the threshold, the HR rose again, while another local pattern of relatively high HR with small *p* value appeared at ELN = 31 (Figure 3a). In the SA cohort, HRs remained below 1 across all tested thresholds. Significant protective effects were observed at several cutoffs, including ELN = 6, 13–15, and 23–29, but the high‐ELN pattern seen in the NACI cohort was not observed (Figure 3b).

### 3.3 Estimation of the Optimal ELN for ESCC Patients

The relationship between ELN count and prognosis suggested distinct patterns according to treatment strategy: in the SA cohort, increasing ELN count was generally associated with continuous survival benefit, whereas in the NACI cohort, both insufficient and excessive lymph node examination appeared to be associated with impaired survival.

**FIGURE 4:**
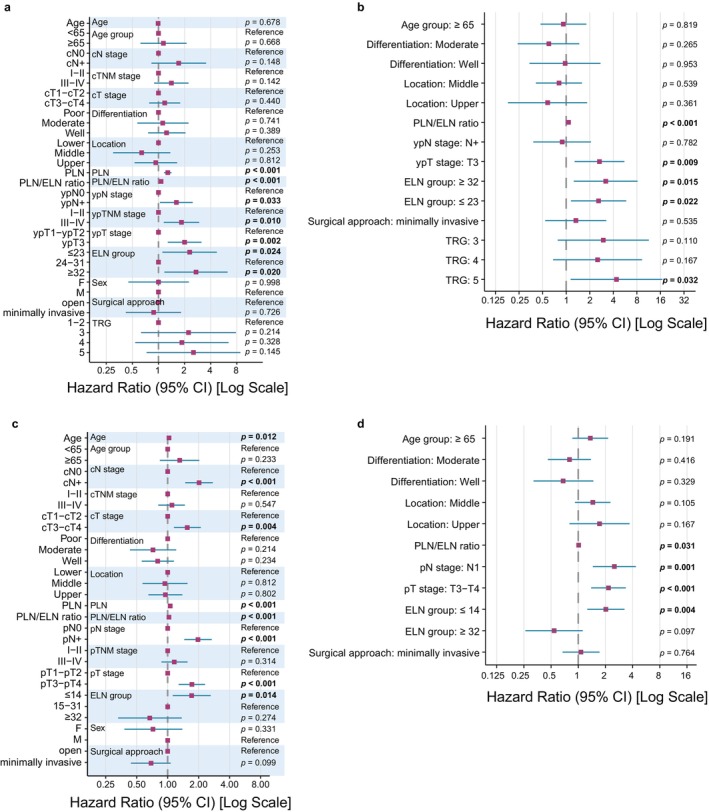
Forest plot of the Cox regression analysis incorporating clinical variables and ELN count groups. (a) NACI group, univariate Cox analysis; (b) NACI group, multivariate Cox analysis; (c) SA group, univariate Cox analysis; (d) SA group, multivariate Cox analysis. HR (95% CI) and *p* value indicate the HR and significance level for the survival advantage of each variable level relative to the reference level. An HR greater than 1 indicates a higher risk.

Based on the threshold analysis, patients in the matched NACI cohort were divided into three groups: ELN ≤ 23, 24–31, and > 31. Univariable Cox analysis showed that both ELN ≤ 23 and ELN > 31 were associated with worse OS than ELN 24–31 (ELN ≤ 23: HR = 2.29, 95% CI: 1.11–4.71, *p* = 0.024; ELN > 31: HR = 2.71, 95% CI: 1.17–6.26, *p* = 0.020). Higher residual PLN count, higher PLN/ELN ratio, and more advanced ypT and ypN stages were also associated with poorer prognosis (Figure 4a).

In multivariable Cox analysis adjusting for age, tumor location, differentiation, ypT stage, ypN stage, PLN/ELN ratio, surgical approach, and tumor regression grade (TRG), both ELN ≤ 23 and ELN > 31 remained independent adverse prognostic factors (ELN ≤ 23: HR = 2.59, 95% CI: 1.15–5.84, *p* = 0.0216; ELN > 31: HR = 3.20, 95% CI: 1.25–8.17, *p* = 0.0152) (Figure 4b), indicating that the association between ELN grouping and survival was not primarily attributable to differences in other potential confounding clinicopathological variables. Given the known prognostic relevance of treatment response after neoadjuvant therapy, we additionally compared TRG distribution across the three ELN groups and found no significant difference (Figure S1).

In the SA cohort, 14 was selected as one cutoff because it was close to thresholds reported in guidelines and previous studies [4, 11, 12, 14] and showed the lowest *p* value with a relatively smaller HR among nearby candidate cutoffs. Because no further cutoff associated with worse survival was identified, 31 was retained as the second cutoff for comparison with the NACI cohort. To assess the robustness of this grouping strategy, we further performed a sensitivity analysis using 12 alternative cutoff combinations around these two thresholds. Across these alternative grouping schemes, the Low group consistently showed HRs > 1, whereas the High group consistently showed HRs < 1, supporting the robustness of the overall prognostic pattern observed in the SA cohort (Figure S2).

Univariate Cox analysis showed that older age, more advanced cT, cN, pT, and pN stages, higher PLN count, and a higher PLN/ELN ratio were significant risk factors. ELN ≤ 14 and ELN > 31 showed contrasting prognostic trends: ELN ≤ 14 was significantly associated with worse survival, whereas ELN > 31 showed a nonsignificant trend toward better survival (ELN ≤ 14: HR = 1.71, 95% CI: 1.12–2.62, *p* = 0.0137; ELN > 31: HR = 0.67, 95% CI: 0.33–1.37, *p* = 0.2737) (Figure 4c). These opposite trends remained in multivariable Cox analysis (ELN ≤ 14: HR = 2.02, 95% CI: 1.26–3.25, *p* = 0.0035; ELN > 31: HR = 0.54, 95% CI: 0.26–1.12, *p* = 0.0967) (Figure 4d).

### 3.4 Subgroup Analyses According to Pathological Nodal Status Further Clarify the Prognostic Role of ELN Count

**FIGURE 5:**
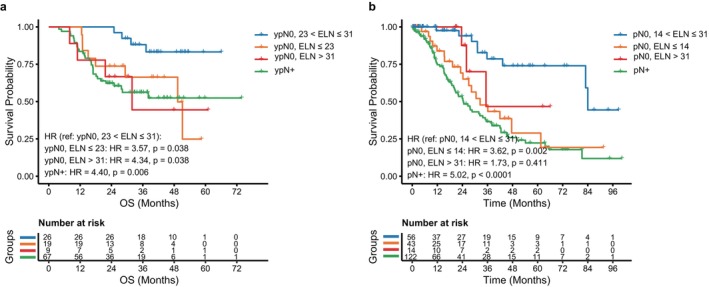
Kaplan–Meier curves for OS of p/ypN0 patients grouped by ELN count. (a) NACI cohort; (b) SA cohort.

To explore whether the worse prognosis associated with insufficient ELN count was related to stage migration, we first analyzed matched p/ypN0 patients. Using the predefined ELN thresholds, patients were divided into three groups. In both the NACI and SA cohorts, patients with low ELN counts had significantly worse OS than those with intermediate ELN counts, and their survival approached that of patients with p/ypN+ disease (Figure 5a,b). These findings suggest that when too few lymph nodes are examined, some patients classified as p/ypN0 may actually harbor occult nodal metastases, resulting in N stage migration.

We then examined whether ELN count remained prognostically relevant in patients with pathologically confirmed nodal metastasis. In the SA cohort, higher ELN counts remained associated with better survival in pN+ patients, consistent with the main analysis (Figure S3 a). In contrast, no significant survival difference was observed across ELN groups within the ypN+ subgroup in the NACI cohort (Figure S3 b).

### 3.5 Single‐Cell RNA and TCR Sequencing Suggest Excessive ELN Count Impairs Immune Response After NACI

ELN > 31 was associated with worse survival in the NACI cohort (Figure 4a,b). Among ypN0 patients, those with ELN > 31 had OS comparable to that of ypN+ patients (Figure 5a), a pattern not observed in the SA cohort. Because PSM excluded all ypT0/ypTis cases from the matched NACI cohort, we further examined this subgroup and found that excessive ELN remained associated with worse survival even in patients with otherwise favorable prognosis (Figure S4). Together, these findings suggest that the prognostic effect of ELN differs between the NACI and SA cohorts and may reflect the immunological importance of preserving nonmetastatic lymph nodes after immunotherapy.

**FIGURE 6:**
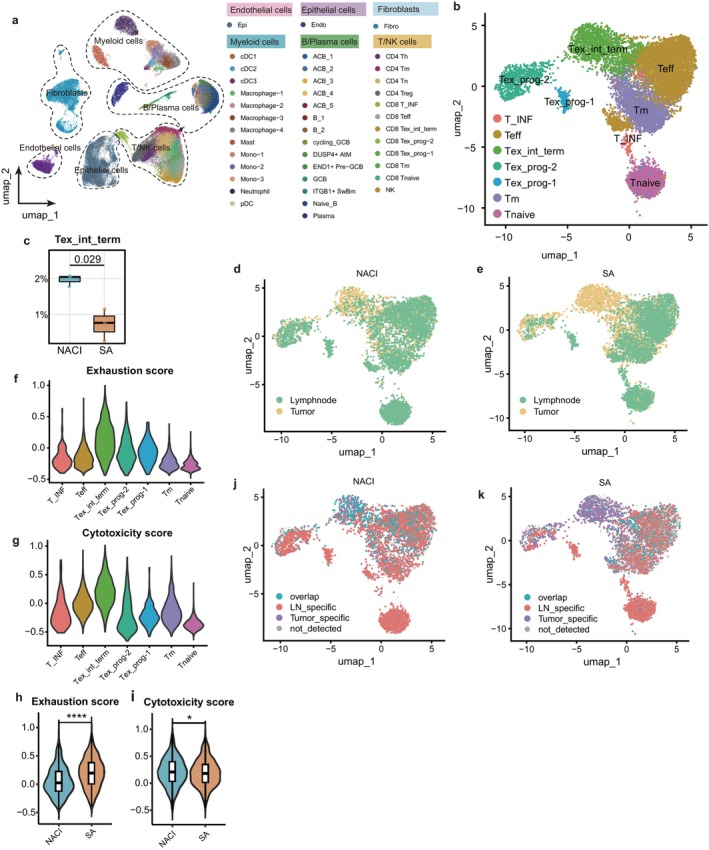
Single‐cell analysis suggests excessive ELN count impairs immune response after NACI. (a) UMAP plot colored by cell annotation; (b) UMAP plot of CD8^+^ T cells colored by cell annotation; (c) Box plots showing the proportion of Tex_int‐term subset among T cells in the NACI and SA groups; (d‐e) UMAP plots of CD8^+^ T cells colored by tissue source (d: NACI group; e: SA group); (f‐g) Violin plots displaying the exhaustion (f) and cytotoxicity (g) scores of each CD8^+^ T cell subset; (h‐i) Violin plots showing the exhaustion (h) and cytotoxicity (i) scores of the Tex_int‐term subset in the NACI and SA groups; (j‐k) UMAP plots colored by the tissue source of CD8^+^ T cell clonotypes (j: NACI group; k: SA group). Figure (c) was analyzed using the *t*‐test, whereas figures (h) and (i) were analyzed using the adjusted Wilcoxon test. *p < 0.05, ****p < 0.0001.

To characterize immune alterations after NACI, we performed scRNA‐seq and single‐cell TCR sequencing on paired tumor tissues and metastasis‐negative lymph nodes from patients treated with SA or NACI. After quality control, 107 199 cells were retained for analysis. Based on unsupervised clustering and canonical markers, we identified major cell types, including B/plasma cells, T/NK cells, myeloid cells, fibroblasts, endothelial cells, and epithelial cells (Figure 6a; Figure S5 a), and further annotated lymphocyte and myeloid subsets according to previous studies [16, 17, 18, 19] (Figure 6b; Figure S5 b–d). Comparison of lymph node composition between treatment groups revealed differences in activated B cell subset 1 (ACB_1), germinal center B cells (GCB), and intermediate/terminally exhausted T cells (Tex_int‐term) (Figure 6c; Figure S6). Tex_int‐term cells were mainly enriched in tumors in the SA group but increased markedly in lymph nodes after NACI (Figure 6d,e). These cells, which have been widely reported as a crucial CD8^+^ T cell subset responsive to ICB therapy [20, 21, 22], showed high exhaustion and cytotoxicity scores (Figure 6f,g), and in the NACI group they exhibited lower exhaustion and higher cytotoxicity than in the SA group (Figure 6h,i).

TCR analysis further identified substantial clonotype sharing between tumors and lymph nodes after NACI, particularly within Tex_int‐term cells (Figure 6j,k), suggesting expansion of tumor‐reactive T‐cell clones in both compartments. Together, these findings suggest persistent immune activity in nonmetastatic lymph nodes after NACI and provide a potential explanation for the adverse prognostic association of very high ELN counts.

## 4 Discussion

While the critical roles of lymph node metastasis in prognosis and of adequate ELN counts in preventing stage migration are well‐established in ESCC, the optimal ELN threshold—particularly in the context of NACI—remains controversial, with significant variations across studies [4, 11, 12, 13].

Our findings align with growing concerns that ICIs challenge the traditional paradigm of extensive lymph node dissection [6]. In our cohort, the postoperative pathological stage of patients receiving NACI was significantly earlier than that of patients undergoing SA, which markedly differed from the baseline status assessed before treatment. Our data suggest that the necessity of extensive lymphadenectomy after NACI should be reconsidered, supporting a more tailored surgical approach.

This retrospective study revealed that ELN count significantly influenced the survival of patients with ESCC. By incorporating groups stratified by ELN count into the Cox regression analysis, we identified two thresholds in the NACI cohort: ELN = 23 and ELN = 31. Survival was significantly compromised when the ELN count was below 23 or exceeded 31, compared to patients whose ELN count fell between these two thresholds. After NACI, a substantial proportion of patients achieved “pathological downstaging”. When lymph node metastases have already responded to therapy, extending the dissection range may no longer provide additional survival benefit [13]. Conversely, survival was impaired when the ELN count exceeded 31 in patients receiving neoadjuvant immunotherapy. This phenomenon might stem from the excessive removal of metastasis‐negative lymph nodes, as our analysis indicated that metastasis‐negative lymph nodes were actively engaged in tumor‐reactive immune effects in response to ICB therapy. Therefore, for patients receiving NACI, it is necessary to intraoperatively balance the benefit of retrieving a sufficient number of lymph nodes against the potential impairment of antitumor immunity associated with an extended dissection range. However, patients undergoing SA appear to derive continuous benefits from an increasing ELN count, although some studies suggest that an excessively extensive surgical scope increases the risk of complications, thereby adversely affecting patient prognosis [23, 24, 25]. This discrepancy might be because the extent of surgery in our center generally did not reach a level that substantially increased surgery‐related complications. Nevertheless, this highlights the need for caution in clinical practice to avoid exceeding an appropriate extent of dissection. Additional subgroup analyses according to pathological nodal status further supported the view that the prognostic role of ELN is context‐dependent and varies according to postoperative nodal status. Among p/ypN0 patients, insufficient ELN examination was associated with significantly worse survival in both cohorts, and their survival approached that of patients with p/ypN+ disease, supporting the possibility of stage migration when nodal assessment is inadequate. However, the prognostic role of ELN differed in node‐positive patients according to treatment context. In the SA cohort, higher ELN counts remained associated with improved survival even among pN+ patients, suggesting that more extensive lymph node examination may still provide prognostic benefit in the presence of pathologically confirmed nodal metastasis. In contrast, no significant survival difference was observed among ELN groups within the ypN+ subgroup of the NACI cohort. This finding suggests that once residual nodal metastasis persists after NACI, prognosis may be driven predominantly by residual treatment‐refractory nodal disease itself, and the adverse biological significance of persistent nodal involvement may outweigh the additional prognostic stratification provided by ELN count.

In summary, our findings highlight the need to carefully tailor the extent of lymphadenectomy in clinical practice, particularly for patients receiving neoadjuvant immunotherapy: it is essential not only to avoid impairing prognosis through excessive anatomical resection but also to maximize the preservation of immune function. In the PRADO trial for melanoma, studies have confirmed that for patients who achieve a major pathological response after neoadjuvant immunotherapy, therapeutic lymph node dissection can be safely omitted without affecting efficacy [26, 27]. A growing number of studies are also focusing on exploring precise preoperative prediction and intraoperative localization of metastasis‐positive lymph nodes [28, 29, 30]. Therefore, future surgical decision‐making requires greater consideration of the treatment context to achieve precise, function‐preserving, individualized surgery.

This study has several limitations. As a retrospective study, it may be subject to selection bias. The case sources were relatively homogeneous, and residual variations in the extent of dissection and in surgeons' technical proficiency may have influenced the results. Furthermore, different methods used by pathologists to retrieve lymph nodes could affect the accuracy of the total lymph node count. Additionally, the thresholds we reported are more statistically derived, and it is challenging to establish precise, universally applicable thresholds. Future multicenter, large‐sample prospective studies are necessary to explore the most suitable extent of ELN dissection for different patient populations.

## 5 Conclusions

Our study suggests that the optimal extent of lymphadenectomy for ESCC is critically influenced by treatment strategy. For patients receiving NACI, an ELN count between 24 and 31 achieves an ideal balance—preventing stage migration while preserving antitumor immunity, as excessive dissection may remove immunologically active sites. In contrast, SA derives continuous survival benefit from higher ELN counts. These findings highlight the imperative for personalized, function‐preserving surgical approaches in the immunotherapy era, urging future validation through prospective multicenter studies.

## Author Contributions

**Kunheng Du:** writing – original draft, writing – review and editing, conceptualization, investigation, validation, methodology, software, formal analysis, visualization, project administration, data curation. **Chenyi Xie:** funding acquisition, writing – review and editing, methodology, data curation, supervision. **Ziyu Ning:** data curation, writing – review and editing. **Hongwei Lin:** conceptualization, investigation, methodology, software, validation, formal analysis, writing – review and editing, visualization, project administration, data curation. **Jiahui Chen:** data curation, writing – review and editing. **Haiyu Zhou:** data curation, supervision, resources, writing – review and editing. **Yihuai Hu:** funding acquisition, writing – review and editing, project administration, resources, supervision. **Zaiyi Liu:** conceptualization, writing – review and editing, project administration, resources, supervision. **Changhong Liang:** data curation, supervision, resources, writing – review and editing.

## Funding

This work was supported by National Natural Science Foundation for Young Scientists, 82202840, 82302299.

## Ethics Statement

All patients signed written informed consent for this study. This study was conducted in accordance with the Declaration of Helsinki and all of the applicable local laws and regulations. Approval for the protocol was obtained from ethics committee of Guangdong Provincial People's Hospital (KY2024‐765‐01, approval date: October 17th, 2024).

## Consent

Informed consent was obtained from all individual participants included in the study.

## Conflicts of Interest

The authors declare no conflicts of interest.

## Supporting information


**Figure S1:** Comparison of the TRG composition among different ELN count groups. Fisher's exact test was used.

**Figure S2:** Forest plot of univariable Cox regression based on grouping by different ELN count thresholds. * *p* < 0.05.

**Figure S3:** Kaplan–Meier curves for different ELN count groups among patients with p/ypN+ disease. (a) SA group; (b) NACI group.

**Figure S4:** Kaplan–Meier curves for patients in the NACI cohort with ypT0 and ypTis, grouped by high and low ELN counts (ELN > 21 vs. ELN ≤ 21).

**Figure S5:** Bubble plots showing the expression levels of genes and gene sets used for clustering. The size of the bubbles represents the percentage of cells with an expression level greater than zero; the color indicates the average expression level, with red representing higher levels and blue representing lower levels. (a) Major cell types; (b) Myeloid cells; (c) B cells; (d) T cells. Endo: Endothelial cells; Epi: Epithelial cells; Fibro: Fibroblasts; pDC: Plasmacytoid dendritic cells; cDC: Conventional dendritic cells; Mono: Monocytes; Tex_int_term: Intermediate/terminally exhausted T cells; Treg: Regulatory T cells; Tex_prog‐1: Progenitor exhausted T cells subset 1; Tex_prog‐2: Progenitor exhausted T cells subset 2; NK: Natural killer cells; Tm: Memory T cells; Teff: Effector T cells; T_INF: INF response T cells; Th: T helper cells; Tn: Naïve T cells; GCB: Germinal center B cells; ACB: Activated B cells; AtM: Atypical memory B cells; SwBm: Switched memory B cells.

**Figure S6:** Proportions of various cell types within the lymph nodes. *p < 0.05; ns: not significant. *p* values were determined by the two‐sided unpaired Student's *t* test.

**Data S1:** Supplementary Methods: Detailed methods for single‐cell RNA and V(D)J sequencing, downstream data analysis, and cell type annotation.

## Data Availability

The datasets used and analyzed during the current study are available from the corresponding author on reasonable request.
