# Supplementary material for: Tailoring the Extent of Lymphadenectomy for Esophageal Squamous Cell Carcinoma: Insights From a Comparative Study of Neoadjuvant Chemo‐Immunotherapy and Surgery Cohort
Source: Thorac Cancer. 2026 May 7;17(9):e70297. doi: 10.1111/1759-7714.70297 (PMC13150998; doi:10.1111/1759-7714.70297)
Supplement: Supplementary file 1 — Figure S1: Comparison of the TRG composition among different ELN count groups. Fisher's exact test was used. [file TCA-17-e70297-s004.docx]

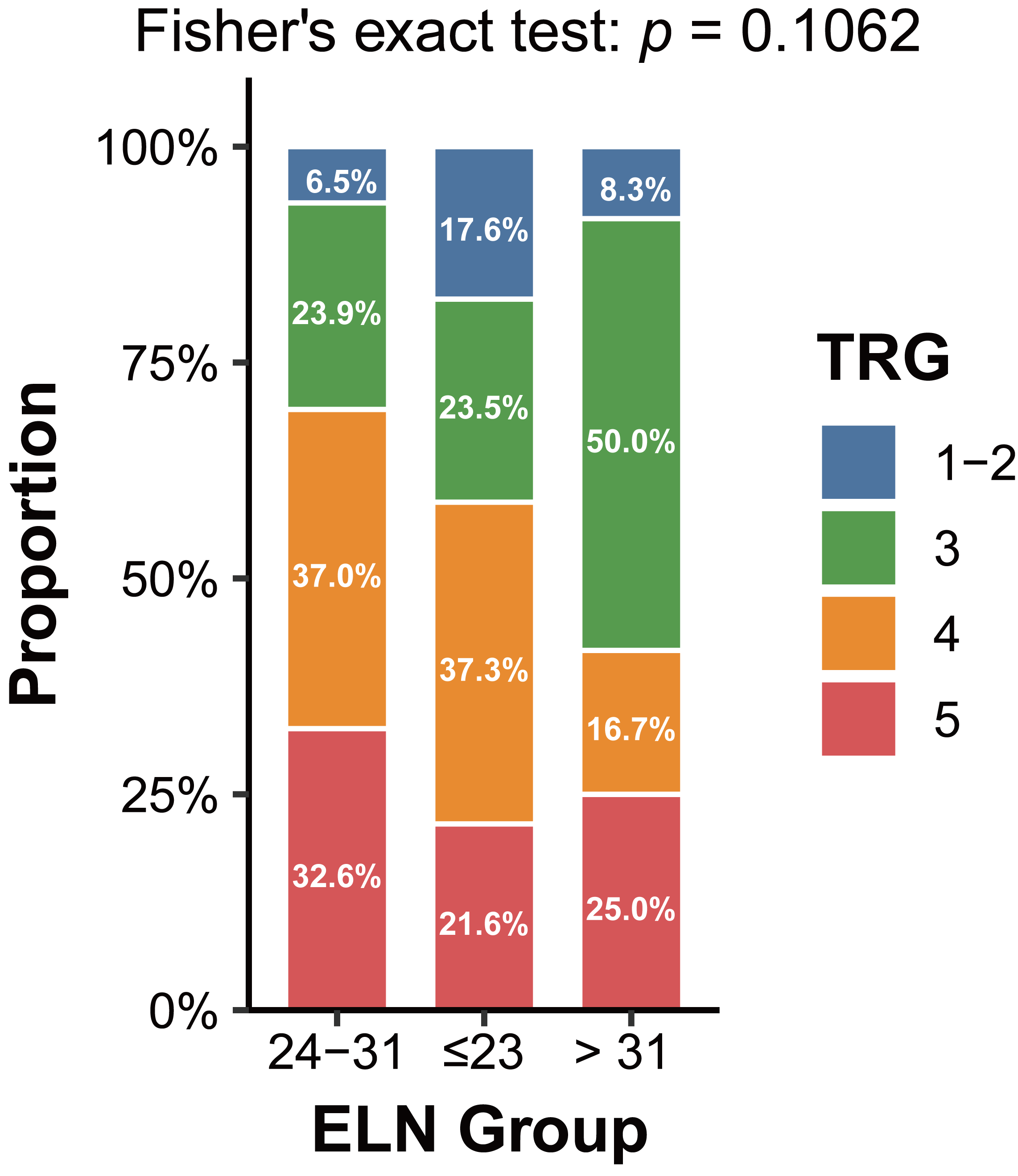


**Figure S1** Comparison of the TRG composition among different ELN count groups. Fisher’s exact test was used.
