# Supplementary material for: Tailoring the Extent of Lymphadenectomy for Esophageal Squamous Cell Carcinoma: Insights From a Comparative Study of Neoadjuvant Chemo‐Immunotherapy and Surgery Cohort
Source: Thorac Cancer. 2026 May 7;17(9):e70297. doi: 10.1111/1759-7714.70297 (PMC13150998; doi:10.1111/1759-7714.70297)
Supplement: Supplementary file 2 — Figure S2: Forest plot of univariable Cox regression based on grouping by different ELN count thresholds. * p < 0.05. [file TCA-17-e70297-s001.docx]

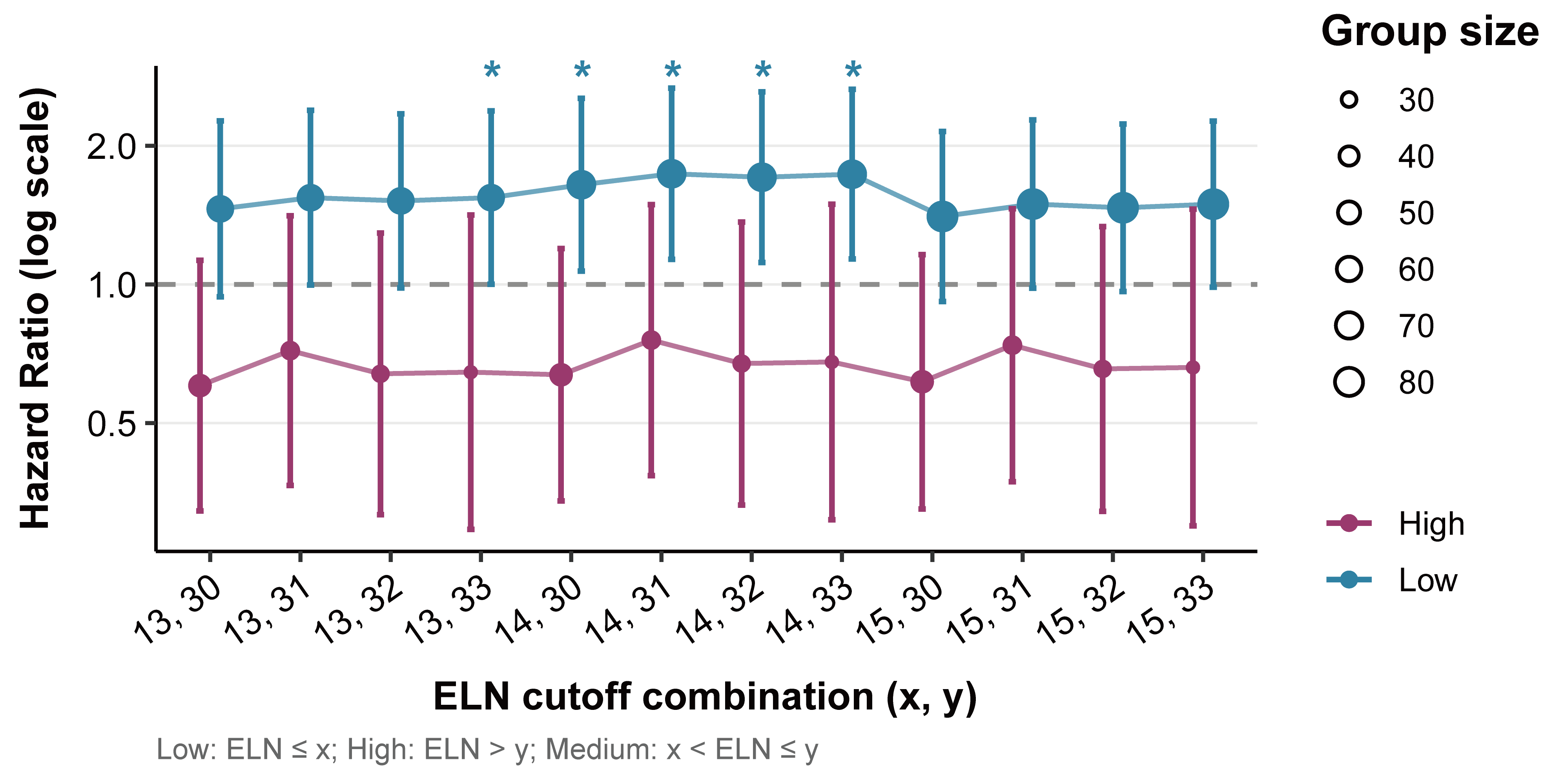


**Figure S2** Forest plot of univariable Cox regression based on grouping by different ELN count thresholds. * *p* < 0.05.
