# Supplementary material for: Tailoring the Extent of Lymphadenectomy for Esophageal Squamous Cell Carcinoma: Insights From a Comparative Study of Neoadjuvant Chemo‐Immunotherapy and Surgery Cohort
Source: Thorac Cancer. 2026 May 7;17(9):e70297. doi: 10.1111/1759-7714.70297 (PMC13150998; doi:10.1111/1759-7714.70297)
Supplement: Supplementary file 3 — Figure S3: Kaplan–Meier curves for different ELN count groups among patients with p/ypN+ disease. (a) SA group; (b) NACI group. [file TCA-17-e70297-s007.docx]

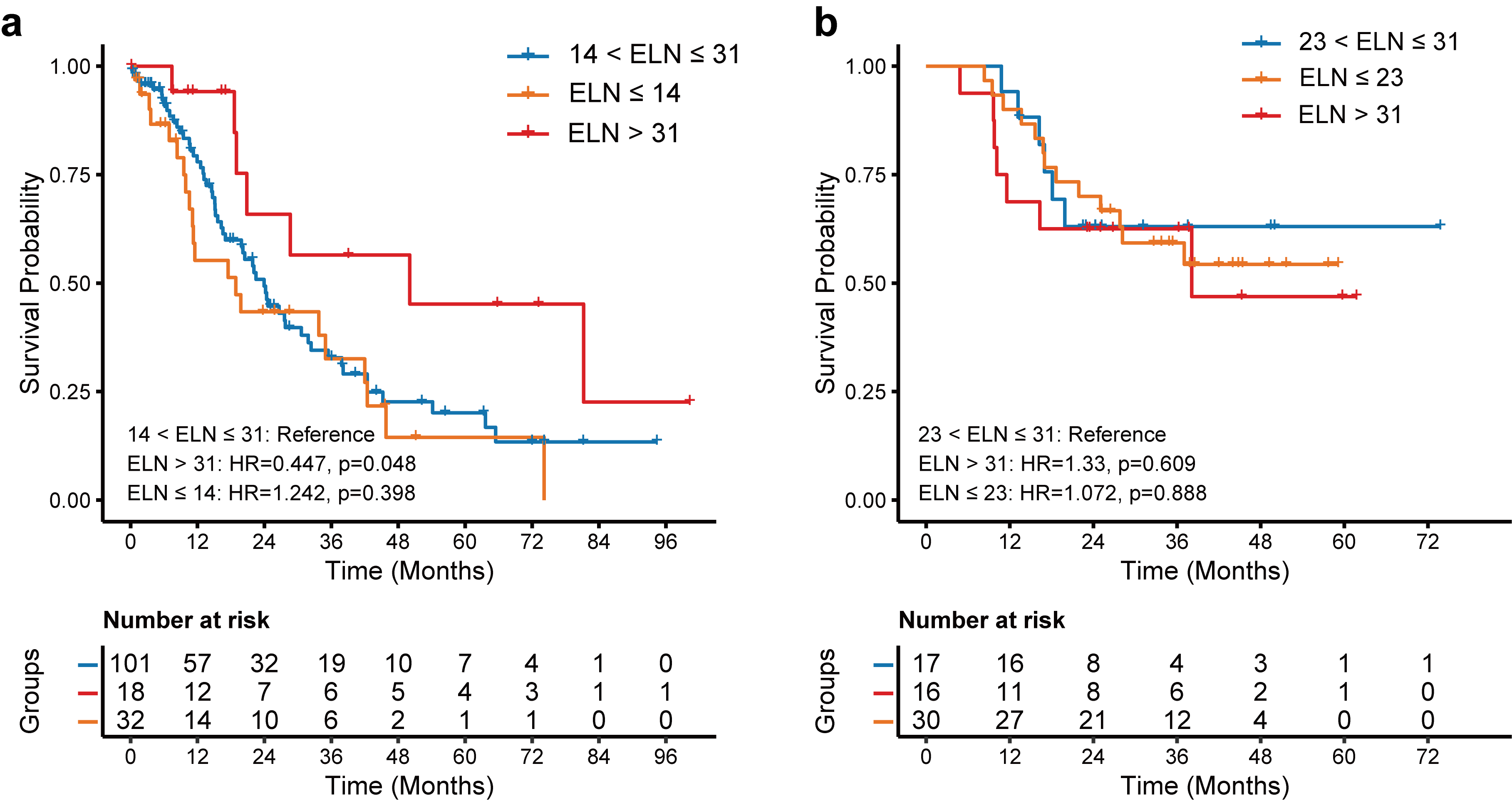


**Figure S3** Kaplan–Meier curves for different ELN count groups among patients with p/ypN+ disease. (a) SA group; (b) NACI group.
