# Supplementary material for: Tailoring the Extent of Lymphadenectomy for Esophageal Squamous Cell Carcinoma: Insights From a Comparative Study of Neoadjuvant Chemo‐Immunotherapy and Surgery Cohort
Source: Thorac Cancer. 2026 May 7;17(9):e70297. doi: 10.1111/1759-7714.70297 (PMC13150998; doi:10.1111/1759-7714.70297)
Supplement: Supplementary file 4 — Figure S4: Kaplan–Meier curves for patients in the NACI cohort with ypT0 and ypTis, grouped by high and low ELN counts (ELN > 21 vs. ELN ≤ 21). [file TCA-17-e70297-s005.docx]

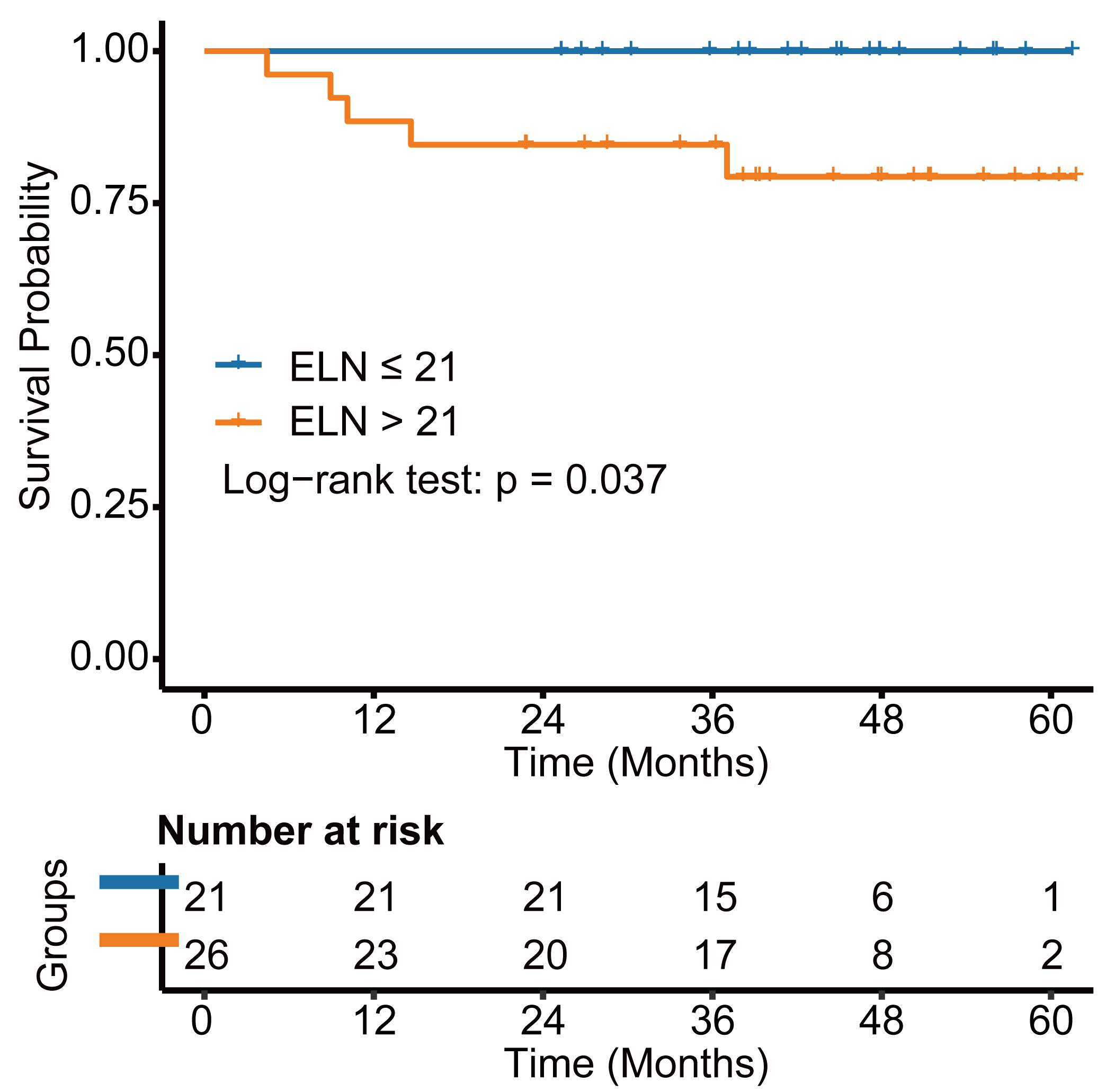


**Figure S4** Kaplan-Meier curves for patients in the NACI cohort with ypT0 and ypTis, grouped by high and low ELN counts (ELN >21 vs. ELN ≤21).
