# Supplementary material for: Tailoring the Extent of Lymphadenectomy for Esophageal Squamous Cell Carcinoma: Insights From a Comparative Study of Neoadjuvant Chemo‐Immunotherapy and Surgery Cohort
Source: Thorac Cancer. 2026 May 7;17(9):e70297. doi: 10.1111/1759-7714.70297 (PMC13150998; doi:10.1111/1759-7714.70297)
Supplement: Supplementary file 5 — Figure S5: Bubble plots showing the expression levels of genes and gene sets used for clustering. The size of the bubbles represents the percentage of cells with an expression level greater than zero; the color indicates the average expression level, with red representing higher levels and blue representing lower levels. (a) Major cell types; (b) Myeloid cells; (c) B cells; (d) T cells. Endo: Endothelial cells; Epi: Epithelial cells; Fibro: Fibroblasts; pDC: Plasmacytoid dendritic cells; cDC: Conventional dendritic cells; Mono: Monocytes; Tex_int_term: Intermediate/terminally exhausted T cells; Treg: Regulatory T cells; Tex_prog‐1: Progenitor exhausted T cells subset 1; Tex_prog‐2: Progenitor exhausted T cells subset 2; NK: Natural killer cells; Tm: Memory T cells; Teff: Effector T cells; T_INF: INF response T cells; Th: T helper cells; Tn: Naïve T cells; GCB: Germinal center B cells; ACB: Activated B cells; AtM: Atypical memory B cells; SwBm: Switched memory B cells. [file TCA-17-e70297-s006.docx]

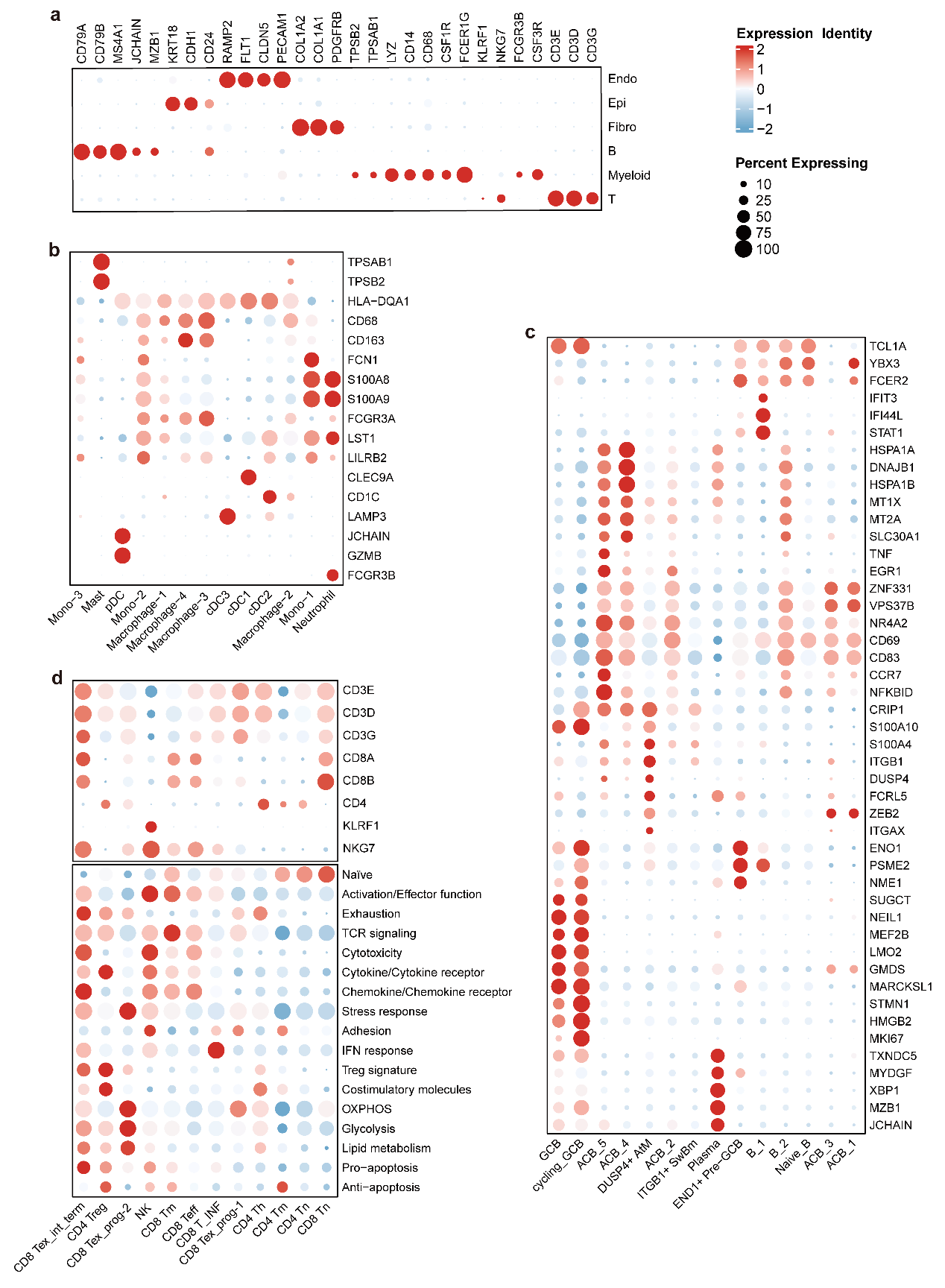


**Figure S5** Bubble plots showing the expression levels of genes and gene sets used for clustering. The size of the bubbles represents the percentage of cells with an expression level greater than zero; the color indicates the average expression level, with red representing higher levels and blue representing lower levels. (a) Major cell types; (b) Myeloid cells; (c) B cells; (d) T cells. Endo: Endothelial cells; Epi: Epithelial cells; Fibro: Fibroblasts; pDC: Plasmacytoid dendritic cells; cDC: Conventional dendritic cells; Mono: Monocytes; Tex_int_term: Intermediate/terminally exhausted T cells; Treg: Regulatory T cells; Tex_prog-1: Progenitor exhausted T cells subset 1; Tex_prog-2: Progenitor exhausted T cells subset 2; NK: Natural killer cells; Tm: Memory T cells; Teff: Effector T cells; T_INF: INF response T cells; Th: T helper cells; Tn: Naïve T cells; GCB: Germinal center B cells; ACB: Activated B cells; AtM: Atypical memory B cells; SwBm: Switched memory B cells.
