# Supplementary material for: Tailoring the Extent of Lymphadenectomy for Esophageal Squamous Cell Carcinoma: Insights From a Comparative Study of Neoadjuvant Chemo‐Immunotherapy and Surgery Cohort
Source: Thorac Cancer. 2026 May 7;17(9):e70297. doi: 10.1111/1759-7714.70297 (PMC13150998; doi:10.1111/1759-7714.70297)
Supplement: Supplementary file 6 — Figure S6: Proportions of various cell types within the lymph nodes. *p < 0.05; ns: not significant. p values were determined by the two‐sided unpaired Student's t test. [file TCA-17-e70297-s003.docx]

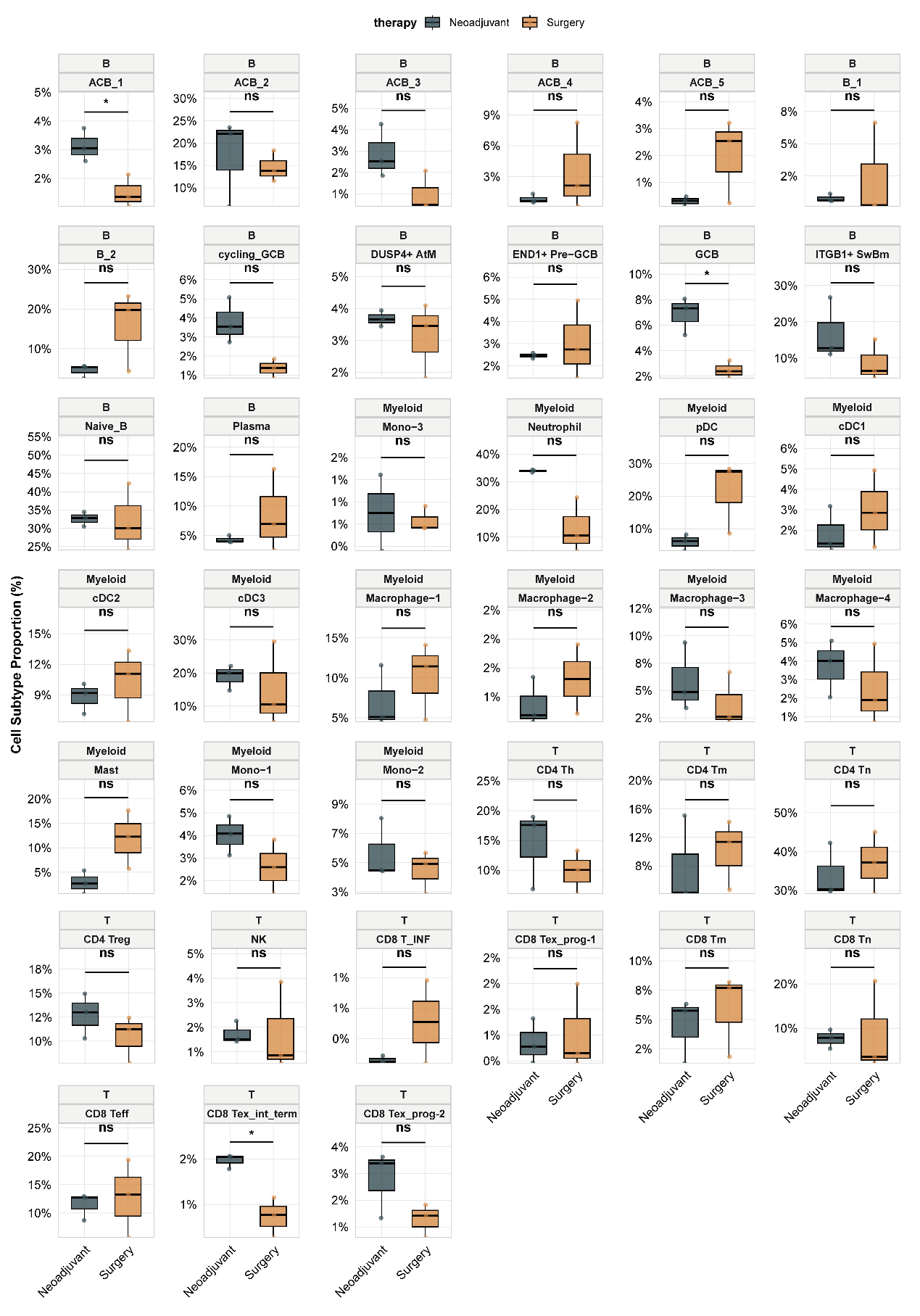


**Figure S6** Proportions of various cell types within the lymph nodes. *: *p* < 0.05; ns: not significant. *P* values were determined by the two-sided unpaired Student’s *t* test.
