# Supplementary material for: Tailoring the Extent of Lymphadenectomy for Esophageal Squamous Cell Carcinoma: Insights From a Comparative Study of Neoadjuvant Chemo‐Immunotherapy and Surgery Cohort
Source: Thorac Cancer. 2026 May 7;17(9):e70297. doi: 10.1111/1759-7714.70297 (PMC13150998; doi:10.1111/1759-7714.70297)
Supplement: Supplementary file 7 — Data S1: Supplementary Methods: Detailed methods for single‐cell RNA and V(D)J sequencing, downstream data analysis, and cell type annotation. [file TCA-17-e70297-s002.docx]

**Single-cell RNA and V(D)J sequencing**

The single cell suspension was adjusted to 700-1200 cells /μL. The 10x Genomics Chromium Next GEM Single Cell 5' Kit v2 (PN-1000263) was used according to the User Guide. Cell suspension and Master Mix, Gel beads and Oil were loaded into a chip to form microdroplets (GEMs), followed by reverse transcription and pre-amplification to generate full-length cDNA. The transcriptome sequencing library was constructed using a 10x Genomics library construction kit (PN-1000190). Using the 10x Genomics single cell V(D)J Enrichment Kit [ TCR (PN-1000005) / BCR (PN-1000016) ] and V(D)J Amplification Kit [ TCR (PN-1000252) / BCR (PN-1000253) ]. The constructed libraries were subjected to high-throughput sequencing using the PE-150 mode at OE Biotech Co. Ltd (Shanghai, China).

**RNA-sequencing data processing**

The Cell Ranger software pipeline (version 3.1.0) provided by 10x Genomics was used to demultiplex cellular barcodes, align reads to the genome and transcriptome using the STAR aligner, and down-sample reads as necessary to generate normalized aggregate data across samples, resulting in a gene count matrix across cells. The unique molecular identifier (UMI) count matrix was processed using the R package Seurat (version 5.1.0)(30). To filter out low-quality cells and likely multiplet captures, a key issue in microdroplet-based experiments, we applied a criterion based on the UMI/gene counts. Specifically, cells with UMI/gene counts outside the range of the 200 to 7000 were excluded, assuming a Gaussian distribution of UMI/gene numbers across cells. In addition, cells exhibiting a high fraction of mitochondrial gene expression (>10%), were also discarded to further remove low-quality cells. Library size normalization was performed in Seurat on the filtered matrix to obtain normalized counts for downstream analysis.

**Cell type annotation**

Top variable genes across single cells were identified by calculating the average expression and dispersion for each gene. Genes were then categorized into expression bins, and principal component analysis (PCA) was performed on the log-transformed gene-barcode matrix of the top variable genes to reduce dimensionality. Cells were clustered using a graph-based clustering approach, and the resulting clusters were visualized in two dimensions using UMAP. To identify marker genes for distinct cell clusters, we employed FindAllMarkers function, comparing cells within a specific cluster to all other cells. Through the use of canonical marker genes, we annotated the cell clusters in the resulting two-dimensional representation with known biological cell types.
